# The Fever Coach Mobile App for Participatory Influenza Surveillance in Children: Usability Study

**DOI:** 10.2196/14276

**Published:** 2019-10-17

**Authors:** Myeongchan Kim, Sehyo Yune, Seyun Chang, Yuseob Jung, Soon Ok Sa, Hyun Wook Han

**Affiliations:** 1 Mobile Doctor, Co, Ltd Seoul Republic of Korea; 2 Institute of Basic Medical Sciences Graduate School of Medicine, CHA University Seongnam-si, Gyeonggi-do Republic of Korea; 3 Department of Biomedical Informatics Graduate School of Medicine, CHA University Seongnam-si, Gyeonggi-do Republic of Korea; 4 Healthcare Bigdata Center, Bundang CHA General Hospital Seongnam-si Republic of Korea

**Keywords:** data collection, detecting epidemics, mobile app, health care app, influenza epidemics, influenza in children

## Abstract

**Background:**

Effective surveillance of influenza requires a broad network of health care providers actively reporting cases of influenza-like illnesses and positive laboratory results. Not only is this traditional surveillance system costly to establish and maintain but there is also a time lag between a change in influenza activity and its detection. A new surveillance system that is both reliable and timely will help public health officials to effectively control an epidemic and mitigate the burden of the disease.

**Objective:**

This study aimed to evaluate the use of parent-reported data of febrile illnesses in children submitted through the Fever Coach app in real-time surveillance of influenza activities.

**Methods:**

Fever Coach is a mobile app designed to help parents and caregivers manage fever in young children, currently mainly serviced in South Korea. The app analyzes data entered by a caregiver and provides tailored information for care of the child based on the child’s age, sex, body weight, body temperature, and accompanying symptoms. Using the data submitted to the app during the 2016-2017 influenza season, we built a regression model that monitors influenza incidence for the 2017-2018 season and validated the model by comparing the predictions with the public influenza surveillance data from the Korea Centers for Disease Control and Prevention (KCDC).

**Results:**

During the 2-year study period, 70,203 diagnosis data, including 7702 influenza reports, were submitted. There was a significant correlation between the influenza activity predicted by Fever Coach and that reported by KCDC (Spearman ρ=0.878; *P*<.001). Using this model, the influenza epidemic in the 2017-2018 season was detected 10 days before the epidemic alert announced by KCDC.

**Conclusions:**

The Fever Coach app successfully collected data from 7.73% (207,699/2,686,580) of the target population by providing care instruction for febrile children. These data were used to develop a model that accurately estimated influenza activity measured by the central government agency using reports from sentinel facilities in the national surveillance network.

## Introduction

Seasonal influenza affects 5% to 15% of the world population and is estimated to be accountable for 3 to 5 million cases of severe illness and up to 500,000 deaths annually [1]. Children younger than 5 years, adults aged 65 years or older, and pregnant women are at high risk of developing serious influenza-related complications [2]. In 2008, it was estimated that 90 million new cases, 1 to 2 million cases of severe illness, and 28,000 to 111,500 deaths that were attributable to influenza infection occurred in this population [3].

Monitoring influenza activities is essential to understand the epidemiology, implement appropriate prevention strategies, and adequately allocate public health resources to mitigate the burden of epidemics. Currently, most developed countries have surveillance systems based on the networks of sentinel facilities and primary care practitioners reporting the weekly number of patients visited with influenza-like illness (ILI) or acute respiratory infection. Many countries, including the United States and South Korea, also collect data of virologic information, hospitalization, and mortality. At a global level, the World Health Organization Global Influenza Surveillance and Response System, comprising 143 National Influenza Centers in 111 countries, has provided virologic information used to select strains for influenza vaccine formulations [4,5].

Although the information provided by influenza surveillance is invaluable, a traditional surveillance system requires substantial amount of resources, including trained staff, laboratory capacities, and information infrastructure. Establishing a sustainable surveillance system in countries with limited resources depends on financial and technical support from external sources as well as active involvement of the national health authority [6]. In addition, although timeliness is a crucial element of infection surveillance, the traditional system is associated with time lags as it takes several days from event to reports, from reports to analysis, and from analysis to dissemination [7].

To overcome the challenges of traditional surveillance systems, novel approaches to influenza surveillance using the internet and mobile technologies have been developed. The use of big data drawn from Web search queries, social media, and electronic health records has attracted much attention from public health researchers and policy makers. Among these, Google Flu Trends (GFT) [8] was one of the key drivers of the hype around big data in public health. Despite its constant overestimations of flu prevalence that led to the termination of the service, GFT shed light on the use of internet big data in disease surveillance and nowcasting [9,10]. A number of researchers have also evaluated search queries and social media as influenza surveillance tools [11-15]. However, the big data approach faces serious challenges including *big data hubris*, privacy concerns, and a lack of accurate algorithmic models [9,10].

Another recently emerged internet-based surveillance method is participatory surveillance [16]. Participatory disease surveillance systems ask the target population to submit disease-related data through various forms of survey tools. Although it is a potentially valuable data source, participatory surveillance has low specificity because many such systems collect subjective symptoms of illness without information on clinical diagnosis [16,17]. Furthermore, as these systems completely rely on volunteers in communities, participation is low and difficult to maintain over time [18].

In this paper, we describe a new approach that collects participatory data, including symptoms and clinical diagnosis, of febrile diseases and encourages participation by providing fever care instructions. In addition to analyzing individual data and delivering customized advice, the vast amount of aggregate information could generate a population-level data. We examined if such data could be used for influenza surveillance while addressing the aforementioned challenges of participatory disease surveillance systems.

## Methods

### Exploratory Data Analysis

#### Participatory Data: Fever Coach

**Figure 1:**
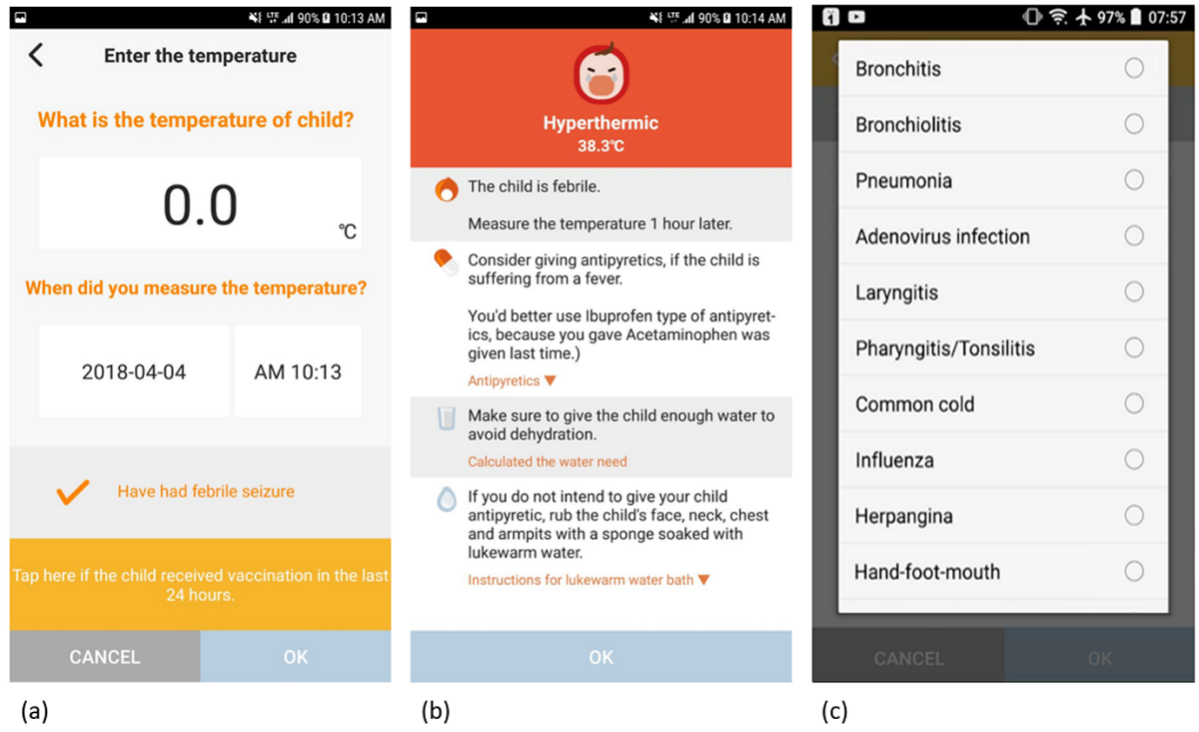
Screenshots of Fever Coach app. (a) body temperature page, (b) fever management page, (c) diagnosis page. The service is not provided in English and is translated from Korean for these screenshots. (options list: bronchitis, bronchiolitis, pneumonia, adenovirus infection, laryngitis, pharyngitis/tonsillitis, common cold, influenza, canker sore, herpangina, hand-foot-mouth, enterocolitis, meningitis, otitis media, urinary tract infection, mumps, scarlet fever, chicken pox, measles, roseola, not diagnosed, and others).

All self-reported data were obtained from Fever Coach. Fever Coach (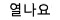 in Korean) is a mobile app created to provide actionable, tailored information for fever management in children younger than 5 years. Upon registration for the service, a user is asked to enter the child’s date of birth, body weight, sex, and history of febrile seizure. A single user can manage multiple accounts if they have more than 1 child. Figure 1 shows screenshots of the app. After registration, the user may enter various fever-related information of the child including body temperature, other symptoms, dose and time of antipyretic administration, vaccination and antibiotic history, and physician-made diagnosis if the child was seen by a physician for the management of current illness. On the basis of these inputs, the app provides instructions for fever control and advises on the timing of seeking medical attention. If a physician-made diagnosis is submitted, detailed information about the disease is also provided. Users can also choose to not submit any data and to only read fever care instructions. The service was designed and reviewed by 2 board-certified family physicians and 1 board-certified pediatrician. Fever Coach was launched in July 2015 in South Korea. The app is currently available for free on Google Play and Apple App Store in South Korea, Japan, and China. As of June 2018, there have been 400,000 downloads, 99% of which were in South Korea.

The study data were collected retrospectively only from users who agreed to the use of deidentified information for research purposes from September 2016 to August 2018. The data submitted between September 2016 and August 2017 (development period, 52 weeks) were used to develop a prediction model, and the data submitted from September 2017 to August 2018 (validation period, 52 weeks) were used to validate the model.

All user-reported data were stored in Fever Coach databases in real time, and metadata (number of submissions and location information) on each day were processed and saved on a daily basis. For comparison with the available reference population, only the data from children aged 1 year (365 days) to 6 years (2554 days) submitted from South Korea were included for this study. To filter out falsely submitted data, we only used body weight values from 5 percentile of newborns (2.5 kg) to 95 percentile of 18 year olds (79.8 kg) based on the Korean child growth curve in 2017 [19]. As the age of a child changed over time, we calculated the mean of the age during each period if data were submitted multiple times. For body weight, we only considered the last submitted data for each child. These data were used to understand user demographics, clinical characteristics, and fever-related behavior. The study protocol was approved by the institutional review board (IRB) of CHA university (IRB number 1044308-201804-HR-022-03).

The total app user activity was measured by the number of body temperature submissions to the app in each week. As Fever Coach is typically used only when a child is ill, the authors decided that it was more appropriate to measure user activity by the number of data submissions than by daily or weekly active users. The proportion of influenza diagnoses to the total number of any diagnoses submitted to the app was used as the indicator of influenza activity.

#### Reference Data: Korea Centers for Disease Control and Prevention Surveillance System

The KCDC ILI surveillance system provides weekly updates on influenza surveillance data as the proportion of the ILI-related visits to the total outpatient visits [20]. An ILI is defined as a fever equal to or greater than 38.0°C (100.4°F) accompanied by cough or sore throat. The surveillance data are collected from the Korea Influenza Surveillance Scheme that has 36 sentinel primary care facilities. KCDC also reports the proportion of the ILI-related visits of children aged 1 year through 6 years to the total outpatient visits of the same age range in a week, as well as in the entire age range.

### Prediction of Influenza Activities

#### Prediction Model

A linear regression model was developed to predict influenza activity in children from the Fever Coach data:

*P* (*t*)=β_0_ + β_1_*F* (*t*) + ε

*P* (*t*) is the proportion of the ILI-related visits of children aged 1 year through 6 years to the total outpatient visits of the same-aged population reported by KCDC, *F* (*t*) and is the proportion of influenza diagnosis reports to all diagnosis reports through the Fever Coach app. Both *P* (*t*) and *F* (*t*) include data accrued in 7 days that precedes date *t* Intercept β_0_ and slope β_1_ were obtained from regression, and ε is an error term. We also developed another linear regression model on *F* (*t*) to predict the proportion of ILI visits in all age ranges to detect the influenza epidemic alert, which is based on the ILI visits in the total population. The threshold of KCDC for an influenza epidemic alert in the 2017 flu season was set at 6.6 ILI visits per 1000 visits, determined as the average value of ILI plus 2 SD during the nonflu seasons in the past 3 years (2014-2016).

#### Statistical Analysis

To compare user characteristics and behavior between the development period and the validation period, the Pearson chi-square test was used for categorical values, the Student *t* test was used for continuous values with normal distribution, and the Mann-Whitney U test was used for continuous values with non-normal distribution. The Shapiro-Wilk test was used for determining the normality of data distribution.

The accuracy of the prediction model was evaluated using the Spearman's rank correlation coefficient (Spearman ρ) and root mean square error (RMSE) for comparison between *P* (*t*) and their predicted values. All statistical analyses were performed using *Scikit-learn 0.19.1* [21].

## Results

### Exploratory Data Analysis

During the entire study period, 10,002,512 fever-related health data were collected. The number of total records decreased by 18.5%, whereas the number of children whose data were submitted decreased by 44.2% from the development period to the validation period (Table 1).

**Table 1:** User characteristics and number of datapoints submitted during the development and validation periods.

Unit, category		Statistical items	Development period (9/1/2016-8/31/2017)	Validation period (9/1/2017-8/31/2018)
**Datapoint**		Total records	5,322,827	4,679,685
	Body temperature	Number of records (%)	4,228,606 (79.44)	3,446,488 (73.65)
		Median (IQR^a^)	38.0 (37.5-38.6)	38.0 (37.5-38.6)^b^
	Antipyretics	Number of records (%)	856,210 (16.08)	1,096,443 (23.43)
	Other symptoms	Number of records (%)	125,338 (2.35)	62,024 (1.33)
	Antibiotics	Number of records (%)	38,122 (0.71)	27,841 (0.59)
	Vaccination	Number of records (%)	24,243 (4.55)	10,828 (2.31)
	Diagnosis	Total number	41,578	26,848
		Influenza, n (%)	2,965 (7.13)	5,800 (21.60)^b^
		Noninfluenza, n (%)	38,613 (92.97)	21,048 (78.37)^b^
**Child**		Total number	209,270	116,804
	Number of data submission (per child)	Median (IQR)	12 (4-32)	21 (7-52)^b^
	Age (years)	Median (IQR)	2.0 (1.4-3.1)	2.2 (1.5-3.3)^b^
	Female sex, %	Ratio	49.22	49.26^c^
	Weight (kg)	Median (IQR)	12.0 (10.7-14.6)	13.3 (11.5-16.0)^b^
	Diagnosis submitted	Total number	27,736	18,128
		Influenza, n (%)	2455 (8.85)	5324 (29.37)
		Noninfluenza, n (%)	25,281 (91.15)	12,804 (70.63)

^a^IQR: interquartile range.

^b^Data showed significant difference between the 2 periods with *P*<.001. History of febrile seizure was not collected during the development period.

^c^*P*=.807.

During the development period, 149,329 people used the service at least once, and 115,674 (77.5%) of them used it for more than 2 separate days during the 1-year development period. From the 149,329 users, fever-related information was recorded from 209,270 children, and 49.2% (103,083) of them were female. This was 7.7% of the total population in that age group in South Korea [22]. They submitted 41,578 diagnoses, including 2965 influenza reports. The minimum and maximum values of *F* (*t*) were 0 and 0.495, respectively, and the range of *P* (*t*) was from 0.0037 to 0.0862 in the same period. The influenza activity during the study period is visualized geographically on a web page titled *Fever Coach Flu Visualization* [23]. The video record of this visualization can be found in Multimedia Appendix 1.

**Figure 2:**
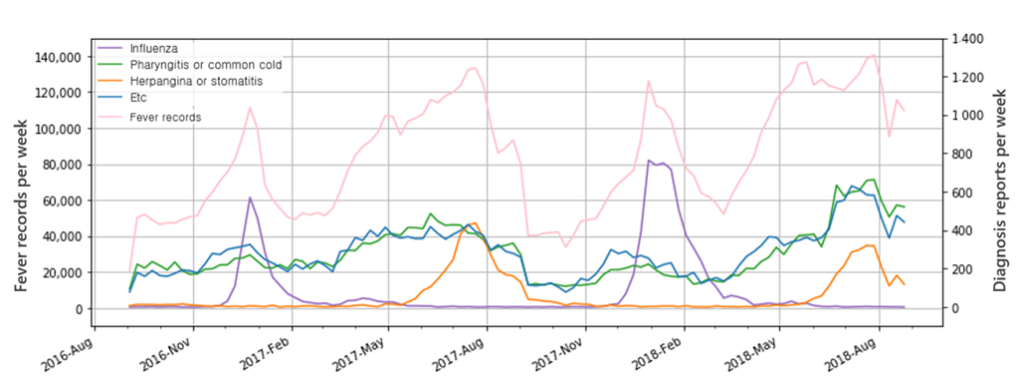
Change of diagnosis submissions and app user activity during the study period. Y-axis shows the number of submissions in each week. Fever records (left y-axis) was measured by the number of body temperature submissions to the app and is shown as a pink line. Number of each diagnosis categories (right y-axis) is shown with different colors. Pharyngitis or common cold (green line) shows the sum of pharyngitis ortonsillitis and common cold, and herpangina or stomatitis (orange line) shows the sum of canker sore and herpangina.

During the validation period, fever-related information of 116,804 children was recorded, and 26,848 diagnoses, including 5800 influenza reports, were collected. *F* (*t*) and *P* (*t*) ranged between 0 to 0.495 and 0.0023 to 0.0928, respectively. We classified all diagnosis records into 3 categories: influenza, pharyngitis/common cold, and herpangina/stomatitis. The change of diagnosis and app user activity over time is shown in Figure 2.

### Prediction of Influenza Activities

**Figure 3:**
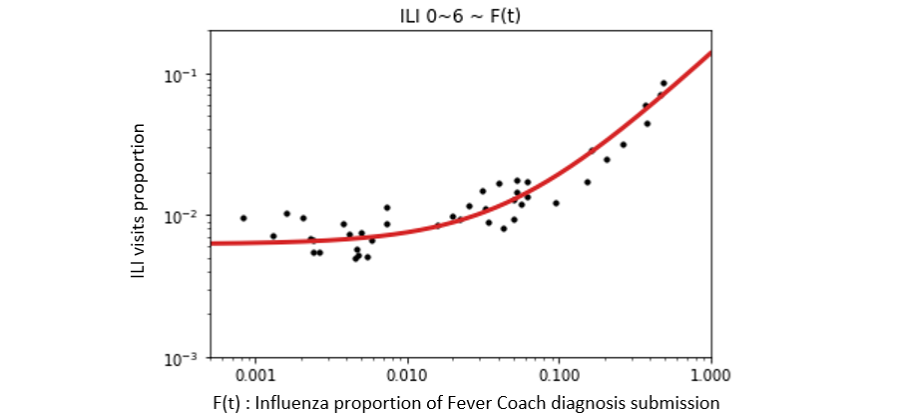
A log-scaled plot. Linear regression between F(t) and influenza-like illness (ILI) visit proportion of children younger than 7 years during the development period. Black dots show actual values, and red line shows the result of linear regression.

**Figure 4:**
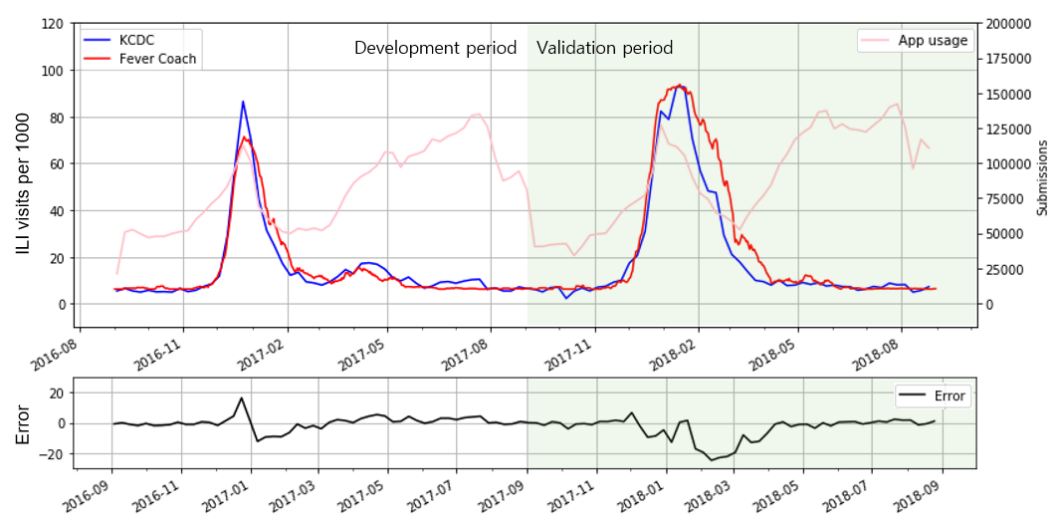
A comparison between Korea Centers for Disease Control and Prevention (KCDC) influenza-like illness (ILI) data and Fever Coach data: blue line shows ILI visits per 1000 outpatient visits reported by KCDC, red line shows ILI prediction from Fever Coach data, and pink line shows the total number of fever-related records in a week. Errors between P (t) and predicted values were displayed at the bottom plot as a black line. The development period was illustrated with a white background color, and green-colored background was used for the validation period.

The prediction model derived from the linear regression between ILI visits [*P* (*t*)] for children aged 1 year through 6 years and influenza diagnosis proportion submitted to Fever Coach [*F* (*t*)] came out with its slope (β_1_) as 0.1316, intercept (β_0_) as 0.0063, and *R*^2^ as 0.930 (Figure 3). *P* (*t*) is the reference value from KCDC. Spearman ρ was 0.895 (*P*<.001) and RMSE was 0.0083 between *P* (*t*) and predicted value in the validation period (the maximum value of *P* (*t*) was 0.0928 and minimum value was 0.0023). Figure 4 shows a graphical plot of *P* (*t*) and predicted values over the study period as well as the error of the prediction model. From the all-age model that was developed between *F* (*t*) and proportion of ILI visits in all age ranges, the 𝜌 was 0.924 (*P*<.001) and the RMSE was 0.0135. The predicted value exceeded the epidemic threshold on November 21, 2017, by this model. KCDC announced the influenza epidemic alert on December 1, 2017.

## Discussion

### Principal Findings

The Fever Coach app successfully collected data from 7.7% of the target population over a 1-year period, which is remarkably higher than participation rates of previously reported Web- or mobile-based participatory surveillance systems that reported participation rates between 0.02% [24] and 0.13% [25]. The influenza activity monitoring model based on voluntary data submitted through the app showed a close association with the large-scale government-reported influenza activity. The participatory data are updated daily, rather than weekly, and the prediction model based on these data led to more sensitive detection of an influenza epidemic compared with the epidemic alert from the KCDC.

The result of this study sheds light on the potential of mobile apps in collecting data for public health surveillance and highlights the importance of user benefits for voluntary data submission. Previous participatory systems for influenza surveillance relied on the social responsibility of individuals for recruiting and retention of participants, which resulted in low participation rate and biased representation of target populations.

### Comparison With Previous Work—Recruitment and Retention

There are several other Web- or mobile phone-based survey platforms for participatory surveillance of influenza and other infectious diseases [17,26,24,27]. Some only collect information regarding symptoms [24] and health-related behavior, whereas others also ask outcomes of medical services [17]. These platforms rely on volunteers by advertising the benefits of self-reported data in disease surveillance and do not incentivize individual participants. Therefore, recruitment and retention of users have been identified as the main challenges for them [24]. They used social media, national television, institutional networks, professional societies, dissemination events, and word of mouth to recruit participants. Using this strategy, Flu Near You (FNY) in the United States recruited 61,000 participants during its second influenza season (2012-2013), which was about 24 per 100,000 population of age 13 years and older. Influenzanet, which was established in 5 European countries in 2009, reported 36,192 participants in 10 countries in Europe during the 2015-2016 influenza season, with a participation rate of 13 individuals per 100,000 population.

In South Korea, online communities are very active and Koreans seek information and communicate with their peers about studying, career, hobbies, and parenting through such communities as well as blogs [28]. Fever Coach used these communities to promote the app by providing free coffee coupons for randomly selected users who downloaded the app and shared their experience and by providing free online health consultation. After reaching 100,000 downloads in May 2016, there have not been active marketing activities, but parents who used the app have voluntarily spread the word on the internet and in their local communities. In February 2017, Fever Coach had been downloaded 300,000 times and over 200,000 users submitted their data at least once. Using this strategy, the participation rate was about 7700 per 100,000 population aged 1 year to 6 years, one of the high-risk groups for influenza infection. The Fever Coach app was primarily designed to enable parents to take care of their kids when they have any kind of fever, but it was able to collect data that can be used specifically for influenza surveillance. We believe that by providing immediate benefit and targeting the vulnerable population, recruiting and retention of participatory surveillance can be much more effective than by relying on volunteers. This strategy can be applied to target other high-risk groups such as pregnant women, elderly people, or immunocompromised individuals.

### Comparison With Previous Work—User Characteristics

As users of Fever Coach submit data on behalf of their children, the only information we collected about the users themselves was email address and device location. However, as the service is designed for caregivers of children younger than 5 years, we can assume that the users’ age range is approximately between late 20s and early 40s, which is younger than the user age ranges reported by other systems. As the app is only accessible via a smartphone, every user is assumed to be a smartphone user. The rate of smartphone ownership in South Korea is reported to be as high as 89.5% [29,30], and the smartphone usage rate is above 99% in the age group between 20 and 50 years [29]. Given the very high rate of smartphone penetration, smartphone users represent the general population of this age group in South Korea. Although there are no data about the gender of the users, it is expected that more females than males use the app based on the higher dependency on mothers for childcare in South Korea. This aligns with other studies that reported female participants being the majority. In a study that analyzed data from FNY, participants reporting for household members showed higher participation rate. This finding can partially explain the successful user recruitment and retention of Fever Coach, which is mostly used by parents for their children. As the service itself is targeted for children, the data collected from the app can only represent the pediatric population. However, our model from these data was able to predict influenza activity in the total population as well as in the pediatric population. This was because influenza activity in children is very closely related to that in adolescents and adults, but it may not be applicable for monitoring other diseases. Another noteworthy finding was the close prediction of influenza activity using the prediction model developed from the previous year despite several changes in the user population. Compared with the development period, the number of users decreased and the median number of data submission per child increased. This finding suggests that retention of active users can make up for the loss of inactive users for participatory surveillance.

### Limitations

There are many limitations of this study that need to be addressed. First, most of the data submitted to Fever Coach came from children with febrile illness, and users generally do not use the app when their children are well. Therefore, increased app usage can either indicate an outbreak of an infectious disease or a surge of interest in the app itself following national media coverage. To overcome this limitation, we only used *diagnosis reports* to estimate ILI visits, contrary to other participatory surveillance systems that ask symptoms of users and use the data for syndromic surveillance. Although this strategy led to a good approximation of influenza activity, it introduces limited generalization. In most regions of South Korea, with physician density of 2.3 per 1000 population [31], primary care is considered affordable and readily accessible. However, in areas where health care is not as accessible, use of physician-made diagnosis may result in a bias toward a small group of people who have better access to health care than the general population. To overcome this second limitation, an additional study to develop more complex prediction models that include symptom and demographic data is in progress.

### Conclusions

The Fever Coach app successfully collected data from 7.73% (207,699/2,686,580) of the target population by providing care instruction for febrile children. These data were used to develop a model that accurately estimated influenza activity measured by the central government agency using reports from sentinel facilities in the national surveillance network.

## Appendix

Multimedia Appendix 1 A video of visualization. Visualization shows diagnosis report submission on the map geographically as time passes.

## Abbreviations

FNY: Flu Near You
GFT: Google Flu Trends
ILI: influenza-like illness
IRB: institutional review board
KCDC: Korea Centers for Disease Control and Prevention
NRF: National Research Foundation of Korea
RMSE: root mean square error
